# Is oesophageal manometry a must before laparoscopic fundoplication? Analysis of 46 consecutive patients treated without preoperative manometry

**DOI:** 10.4103/0972-9941.68581

**Published:** 2010

**Authors:** Anish P Nagpal, Harshad Soni, Sanjiv Haribhakti

**Affiliations:** Department of Surgical Gastroenterology, Haribhakti Surgical Hospital, Ahmedabad, India

**Keywords:** Gastroesophageal reflux disease, laparoscopic Nissen’s fundoplication, oesophageal manometry, outcomes

## Abstract

**AIMS:**

To evaluate retrospectively the outcome of laparoscopic fundoplication in a cohort of patients with typical symptoms of gastroesophageal reflux disease (GERD).

**MATERIALS AND METHODS:**

Forty-six patients with typical symptoms of GERD, from March 2001 to November 2009, were studied. The study was limited to patients with positive findings on upper GI endoscopy done by ourselves and “typical” symptoms (heartburn, regurgitation, and dysphagia) of GERD. Laparoscopic Nissen’s fundoplication was performed when clinical assessment suggested adequate oesophageal motility and length. Only 1 patient, who had negative endoscopic findings, underwent a 24-hour pH-monitoring before surgery. Outcome measures included assessment of the relief of the primary symptom responsible for surgery in the early postoperative period; the patient’s evaluation of outcome and quality of life after surgery.

**RESULTS:**

Relief of the primary symptom responsible for surgery was achieved in 85% of patients at a mean follow-up of 28 months. Thirty-nine patients were asymptomatic, 2 had minor gastrointestinal symptoms not requiring medical therapy, 3 patients had gastrointestinal symptoms requiring medical therapy/ Proton Pump Inhibitors and in 2 patients the symptoms worsened after surgery. There were no deaths. Clinically significant complications occurred in 6 patients. Median hospital stay was 3 days, decreasing from 6 in the first 10 patients to 3 in the last 10 patients.

**CONCLUSIONS:**

Preoperative oesophageal manometry is not mandatory for laparoscopic fundoplication done in selected patients with typical symptoms of GERD and upper GI endoscopy suggestive of large hiatus hernia.

## INTRODUCTION

The development of laparoscopic fundoplication over the past several years has resulted in renewed interest in the surgical treatment of gastroesophageal reflux disease (GERD). Historically, effective treatment options for GERD have included lifelong antireflux medication and antireflux surgery. Laparoscopic fundoplication (LF) has been shown to be safe and effective for the treatment of GERD, with 90% to 94% overall patient satisfaction at long-term follow-up.[1–3] In the appropriate clinical setting, the diagnosis of GERD relies on the demonstration of ONE of the following:

1. The presence of documented (photographic or histologic) oesophageal mucosal injury (oesophagitis) or
2. excessive acid reflux during 24-hour intra-oesophageal pH-monitoring

Additional studies may be used for confirmation in difficult cases (e.g., contrast radiographic studies, symptom-mapping with provocative tests, gastric emptying studies).[4]

Quality of life analyses have become an important part of surgical outcome analyses. Disease-specific questionnaires have been used in an attempt to quantify quality of life before and after medical intervention. This study was done to evaluate retrospectively the outcome of laparoscopic fundoplication in a cohort of patients operated by us with typical symptoms of GERD without using manometry.

## MATERIALS AND METHODS

Forty-six patients with typical symptoms of GERD who were operated for laparoscopic Nissen’s fundoplication from March 2001 to November 2009 were studied. Mean age was 49.7 years, and the oldest patient was 78 years old. There were 26 (56.5%) male and 20 (43.5%) female patients. The most common symptom was heartburn, followed by regurgitation and constipation. Most patients (34/46) were taking proton pump inhibitors for acid suppression and either had breakthrough symptoms or more commonly desired an alternative to lifelong medication. Failure of medical therapy was not considered as an indication for fundoplication, and all patients were offered the alternatives of continuing with medical therapy or undergoing antireflux surgery. The study was limited to patients with positive findings on upper GI endoscopy done by ourselves and “typical” symptoms (heartburn, regurgitation, and dysphagia) of GERD. Laparoscopic fundoplication was performed when clinical assessment suggested adequate oesophageal motility and length. The indications for surgery were (1) complications of GERD, viz., oesophageal stricture (n= 4) and Barrett’s oesophagus (n= 2); (2) large (>5 cm) sliding or para-oesophageal hiatal hernia (n= 4); (3) patient’s desire to discontinue medical treatment that was controlling reflux oesophagitis (n= 36). Out of these 36 patients, hiatus hernia (3 to 5 cm) was present in 29 patients.

All patients underwent oesophagogastroduodenoscopy prior to surgery. Oesophageal manometry was not done in any patient due to unavailability. Twenty-four–hour oesophageal pH-monitoring was performed for only 1 patient, who had negative endoscopic findings but had severe GERD symptoms. All patients were admitted to the hospital on the day of operation. Antibiotics were not used routinely.

### Operative procedure

The procedure is carried out using general anaesthesia with the patient in the lithotomy (or legs apart) position and reverse Trendelenburg position. The surgeon stands in between the legs, and the assistant holding the camera stands on the right of the patient; and the other assisting surgeon, on the left. Pneumoperitoneum is produced using a veress needle introduced through a small supraumbilical incision. After placing all trocars, the first step of the procedure is to separate the herniated stomach from the hiatus. Later we start with the division of the hepatic omentum along the upper lesser curvature of the stomach. This should be done over a short distance to avoid damage to the hepatic branches of the vagus nerve.

The next step is to retract the stomach to the left and expose the right crus of the diaphragm. The stomach is retracted to the right, and the left crus is identified. The oesophagus is elevated, and the posterior vagus nerve can be identified easily behind the oesophagus. Both are lifted, and posterior window is created. Great care should be exercised in making this opening under clear vision without damaging the posterior wall of the stomach or oesophagus. An umbilical tape is passed posterior to the oesophagus to help in retraction. A complete dissection of the lateral and inferior aspects of the left crus and fundus of the stomach is the key manoeuvre allowing circumferential mobilization of the oesophagus.

The left and right crura should be stripped of their surface connective tissue in preparation for the crural closure behind the oesophagus. This usually is accomplished using two to three 2-0 Prolene sutures passed through the muscle bundles of the crura. The hiatus is closed, taking care not to snugly tighten it. No bougie is used to close the hiatal opening. The fundus of the stomach is mobilised, and about 2 or 3 upper short gastric vessels are divided using the ultrasonic scalpel. With caution and meticulous dissection, the fundus can be completely mobilised in almost all patients.

This dissection is done till the fat near the angle of His is reached. The fundus is taken posterior to the lower oesophagus, and a loose wrap is made taking care that the anterior wall of the fundus is brought over the anterior wall of the oesophagus above the supporting umbilical tape. Avoid twisting the gastric fundus around the oesophagus by ensuring that the posterior fundus is used in the construction of the fundoplication — it is grasped and passed behind the oesophagus from left to right rather than pulled from right to left. The anterior and posterior lips of the fundoplication are sutured together using two or three Prolene 2-0 sutures, with at least one suture passing through the anterior oesophageal wall. The wrap created is also fixed to the right crus with one stitch of silk 2-0 or Prolene 2-0.

Nissen’s fundoplication was performed in all but one patient, in whom the procedure was converted to a Toupet procedure. No patient had previously undergone fundoplication or an oesophageal operation. A diet of clear liquids was begun next day after the operation, and the diet was advanced as tolerated. Individuals were instructed to chew the food well and to eat small meals. Gastrograffin study was not done routinely. All patients were discharged on the second or third postoperative day. In 1 patient with large para esophageal hernia, there was bleeding from the left hepatic vein, which required conversion to open procedure. The patient’s postoperative recovery was uneventful.

### Follow-up

Follow-up time ranged from 1 month to 7 years, with an average of 28 months. Data on operative time, period of hospitalization, and complications were collected for all patients. Outcome measures included assessment of the relief of the primary symptom responsible for surgery in the early postoperative period; the patient’s evaluation of outcome and quality of life after surgery. Out of the 46 patients, 30 patients were on regular follow-up on an outpatient basis, and the remaining 16 were asked to complete a questionnaire by telephonic conversation. Tables 1–4 show the questionnaire format.

**Table 1:** Side effects of the operation

Side Effect	Number of Patients
Temporary swallowing discomfort	1
Dysphagia >3 months	2
Complaints of bloating	4
Inability to belch	None

**Table 2:** Antireflux medication before and after surgery

	Preoperative	Postoperative
PPI	34	4
H2 blockers	10	2
Antacids	2	-
Calcium channel blockers	-	1
Anti-anxiety drugs	-	1
Prokinetics	-	1
None	-	37

**Table 3:** Patients’ assessment of outcome

Status/ Quality of life evaluation	Number of Patients
Cured/ excellent outcome: Asymptomatic	39
Improved/ good outcome: Relief of primary symptom, but minor GI symptoms persist which required no therapy	2
Satisfied with surgery: Average outcome Improved, persistent GERD symptoms, therapy required	3
Poor/ worsened: Not improved and/ or long-term dysphagia as a consequence of surgical therapy	2

**Table 4:** Disease-specific questions

Questionnaire asking whether following were experienced	Number of patients answering ‘yes’
Abdominal pain	None
Heartburn	4
Dysphagia	3
Reflux	3
Constipation	5
Bloating	1

## RESULTS

Relief of the primary symptom responsible for surgery was achieved in 85% of patients at a mean follow-up of 28 months. Thirty-nine patients were asymptomatic, 2 had minor gastrointestinal symptoms not requiring medical therapy, 3 had gastrointestinal symptoms requiring medical therapy/ PPI and 2 patients’ symptoms worsened [Tables 2 and 3]. Side effects of the operation are summarized in Table 1. Occasional difficulty swallowing not present before surgery occurred in 2 patients at 6 months after surgery. Temporary swallowing difficulty was seen in 1 patient; and complaint of bloating sensation, in 4 patients. There were no deaths. Clinically significant complications occurred in 6 patients. Median hospital stay was 3 days, decreasing from 6 in the first 10 patients to 3 in the last 10 patients.

### Postoperative complications

One patient had massive pulmonary embolism on the fifth postoperative day (POD) but survived. Two patients had severe port-side infection, for which debridement was done in 1 patient and the other patient had atypical mycobacterium infection treated by Anti Koch’s Treatment AKT. In 1 patient, Nissen’s procedure was converted to Toupet procedure laparoscopically after 2 days, due to severe dysphagia. One patient had left pleural effusion, which required ultrasound-guided aspiration.

## DISCUSSION

The advent of the laparoscopic approach provides an ideal opportunity to standardize the technique of Nissen’s fundoplication, because it markedly limits the technical variability that can occur with the open procedure. Heartburn is the classic symptom of GERD.[5] Patients with GERD can be divided into those with “typical” symptoms (heartburn, regurgitation and dysphagia) and those with “atypical” symptoms (cough, hoarseness and wheezing). Dysphagia is reported by more than 30% of individuals with GERD.[6] Typical symptoms are a more reliable and precise guide to the presence of disease, and consequently their improvement better reflects the effectiveness of therapy. For these reasons, we chose to include the disease-specific questionnaire as the basis for the retrospective evaluation of consecutive patients operated using laparoscopic Nissen’s fundoplication technique.

The diagnosis of gastroesophageal reflux disease (GERD) entails the identification of patients with oesophagitis and its complications, as well as patients who have symptoms but no mucosal disease. Endoscopy is mandatory to establish a diagnosis of reflux oesophagitis, to exclude other oesophageal disease and to permit directed biopsy if malignancy is suspected. Measures of oesophageal acid exposure time may be used to quantify reflux before and after treatment; however, if the patient has typical symptoms but no oesophagitis, a temporal association between symptoms and episodes of oesophageal acidification should be sought. Ambulatory 24-hour oesophageal pH-monitoring with accurate event-marking provides recordings suitable for an objective statistical analysis, which was done in only 1 patient, who had typical symptoms of GERD but negative endoscopic findings. Although oesophageal manometry and 24-hour pH-monitoring might be necessary with abnormal findings on videofluoroscopy or atypical symptoms, their routine use is not essential in preoperative evaluation of patients undergoing fundoplication for gastroesophageal reflux disease.[7]

## CONCLUSION

Laparoscopic fundoplication is an effective long-term treatment for GERD and may be performed in patients with typical symptoms of GERD and endoscopic findings suggestive of hiatus hernia. Preoperative oesophageal manometry is not mandatory for laparoscopic fundoplication but is required in selected patients with atypical symptoms.
